# Trends in burden and risk factors associated with childhood stunting in Rwanda from 2000 to 2015: policy and program implications

**DOI:** 10.1186/s12889-020-8164-4

**Published:** 2020-01-20

**Authors:** Agnes Binagwaho, Alphonse Rukundo, Samuel Powers, Kateri B. Donahoe, Mawuena Agbonyitor, Fidel Ngabo, Corine Karema, Kirstin Woody Scott, Mary C. Smith Fawzi

**Affiliations:** 1 University of Global Health Equity, Kigali Heights, Plot 772, KG 7 Ave., 5th Floor, PO Box 6955, Kigali, Rwanda; 2 Harvard Medical School, Boston, MA USA; 3 Dartmouth Geisel School of Medicine, Hanover, NH USA; 4 Independent Consultant, Montréal, Québec, Canada; 5 University of Virginia, Charlottesville, VA USA; 6 Partners In Health, Freetown, Sierra Leone; 7 School of Public Health, Universite libre de Bruxelles, Brussels, Belgium

**Keywords:** Malnutrition, Rwanda, Children, Stunting, Global health

## Abstract

**Background:**

Rwanda has made substantial economic progress over the past two decades. However, evidence suggests that malnutrition among children remains high in spite of this progress. This study aims to examine trends and potential risk factors associated with childhood stunting from 2000 to 2015 in Rwanda.

**Methods:**

Data for this study come from the 2000 to 2015 Rwanda’s Demographic and Health Surveys (DHS), a cross-sectional, population-based survey that is conducted every 5 years. Following prior work, we define stunting based on age and weight as reported in the DHS. We assess the overall prevalence of stunting among children under the age of 5 in Rwanda and then conduct bivariate analyses across a range of policy-relevant demographic, socioeconomic, and health variables. We then incorporate key variables in a multivariable analysis to identify those factors that are independently associated with stunting.

**Results:**

The prevalence of stunting among children under the age of 5 in Rwanda declined from 2000 (47.4%) to 2015 (38.3%), though rates were relatively stagnant between 2000 and 2010. Factors associated with higher rates of stunting included living in the lowest wealth quintile, having a mother with limited education, having a mother that smoked, being of the male sex, and being of low-birth weight.

**Conclusions:**

Though overall stunting rates have improved nationally, these gains have been uneven. Furthering ongoing national policies to address these disparities while also working to reduce the overall risk of malnutrition will be necessary for Rwanda to reach its overall economic and health equity goals.

## Background

Malnutrition is a leading cause of mortality among children around the world, with over 6 million deaths occurring each year [1, 2]. In addition to accounting for more than one-third of child mortality worldwide, the morbidity stemming from malnutrition is of serious consequence as it negatively impacts a child’s future growth and development. Malnutrition is associated with stunting as well as impaired cognitive development, which can result in poor educational outcomes [3, 4]. Longer term, this educational deficit negatively impacts children’s economic security as they transition into adulthood, as well as overall national productivity [5, 6].

Fortunately, chronic malnutrition among children as measured by the resultant effect of stunting has declined globally by an estimated 40 million cases between 1980 and 2000 [7]. However, this progress has not been evenly distributed throughout the world [7]. For instance, some studies have shown an overall modest decline in stunting across Africa, with the overall prevalence decreasing from 40.5% in 1980 to 35.2% in 2000; yet other evidence over this same period demonstrates a rise in stunting within a subset of African countries, including Rwanda, rural Ethiopia, and Côte d’Ivoire, among other countries [7]. The region of East Africa, where Rwanda is situated, witnessed not only the highest rates of stunting but also was the only region to witness a net increase in the prevalence of stunting from 1980 to 2000 (by 0.08% per year) [7].

In Rwanda, the burden of malnutrition may have increased due to the severe political and economic crisis surrounding the 1994 genocide [8]. Since this time, Rwanda has taken a number of progressive steps towards recovery and economic growth as outlined in its Vision 2020 plan which was issued in 2002 [9]. Correspondingly, the health of the population has improved dramatically across an array of population health parameters [10]. For instance, between 2000 and 2015, modern contraceptive use has increased from 3.4 to 27.8% among women of reproductive age, vaccination rates have improved markedly, there has been a 70.1% decline in infant mortality, and a reduction in under-5 mortality from 196 deaths per 1000 live births to 50 deaths per 1000 live births [11]. This progress is related to comprehensive reforms and innovations designed with community engagement to strengthen the health system, including but not limited to some of the following policies: implementing the community-based health insurance plan (known as *Mutuelles de Santé*) to improve financial access to care; developing a robust community health worker network to provide care at the village level; and, creating performance-based financing programs to increase the quality of health care services [12–14].

Despite this national progress in health outcomes, malnutrition remains a serious burden among Rwandan children under the age of five, with the rate of stunting remaining relatively stagnant [15–17]. Given that the persistent burden of stunting conflicts with the overall improvements in health outcomes in the country, this study aims to examine trends and explore potential risk factors associated with childhood stunting from 2000 to 2015 in Rwanda. A greater understanding of the policy-relevant factors associated with enduring childhood stunting will help to inform the development of more effective programs and policies to address this persistent challenge.

## Methods

### Study design

Data for this study come from Rwanda’s Demographic and Health Survey (DHS) between 2000 and 2015 [15, 16, 18, 19]. The DHS is a cross-sectional survey conducted every 5 years by the Rwandan National Institute of Statistics in collaboration with the Ministry of Health. Additional financial and technical support is provided from Macro Measure DHS, non-governmental organizations, and bilateral and multilateral organizations [15, 16, 18, 19].

### Sample

The DHS comprises a nationally representative sample of 9696, 10,272, 12,540, and 12,699 households with a response rate of 99.5, 99.7, 99.8, and 99.9% from 2000, 2005, 2010, and 2015, respectively. Data on anthropometric measurements were collected in 50% of households selected for survey. All women aged 15–49 and children under age 5 were measured. Data on nutritional status were analyzed for 6287, 3765, 4076, and 3538 children respectively, in 2000, 2005, 2010, and 2015 [15, 16, 18, 19].

### Variables

The DHS collects data on a variety of demographic, socioeconomic, and health variables, including maternal and child health, anthropometric measurements, male circumcision rates, domestic violence, expenditures on health care, and ownership and use of anti-malarial bed nets. This study relied upon the standard DHS questions related to feeding practices of infants and young children administrated by trained DHS interviewers as well as anthropometric data systematically measured from the sub-sampled group (e.g., the height, age, and sex of the child) [15, 16, 18, 19].

Child nutritional status was assessed using the 2006 World Health Organisation (WHO) child growth standards across the time series of interest in this study. Following prior work, we classify children as stunted for their age if their height-for-age Z-scores fall below two standard deviations (− 2 SD) away the mean of the reference population; these children would be in the bottom 2.5% percentile for height for their age group [20, 21]. The height measurements were carried out using a Shorr measuring board for infants produced under the guidance of the United Nations International Children’s Emergency Fund (UNICEF). Children younger than 24 months were measured lying down (recumbent length) on the board, whereas older children were measured using standard height [15, 19, 22]. For this analysis, we recalculated the prevalence of stunting in accordance to the 2006 WHO guidelines in order to ensure comparability over time as changes in the definition occurred throughout the time series [16, 18].

In terms of covariates, we captured a number of policy-relevant maternal and individual characteristics that are known to influence stunting. Maternal factors included the following: age, education level, employment, history of smoking, and marital status. Other variables of interest included the child’s birth weight, wealth quintile (which served as a proxy for socioeconomic status), household setting (rural versus urban), and the number of children under 5 years of age in the household. Education level had three categories: no formal education, only primary education, and secondary/post-secondary education. A child’s birth weight was categorized based on medical records and/or maternal recall. Children with a weight less than 2.5 kg were classified as low birth weight. The wealth index variable is compiled by the DHS using information regarding ownership of durable goods (e.g., a television, radio, car, etc.) and housing characteristics (e.g., source of drinking water, type of flooring material, access to electricity, etc.) and adjusts for area-specific (urban versus rural) factors scores to create a nationally-applicable combined wealth index. National wealth quintiles are created by ranking each household member based on his or her assigned index scores and then dividing the population based on these scores into quintiles each comprised of 20% of the population [15, 16, 18, 19, 23]. Though maternal height is a variable captured by the DHS and some prior work has shown it to be associated with stunting [24], other studies have shown mid-parental height as a more useful metric [25]. Further, a measure of parental height was not included in this analysis given both the lack of it being amenable to actional policy measures and it being of a particularly sensitive nature within the Rwandan context.

### Analysis

We provide descriptive statistics for the study sample characteristics. Summary descriptive measures, including proportions with 95% confidence intervals for categorical variables, were estimated. Childhood stunting was assessed by height-for-age Z-scores, based on 2006 WHO Child Growth Standards. We then performed bivariate analyses using Pearson chi-squared tests to examine the association between stunting in children and a number of independent predictor variables including household, maternal, and child variables. The choice of the independent variables was based on variables collected and available in the DHS data sets and on the literature reviews on the socio-demographic characteristics influencing childhood stunting. Finally, we used logistic regression to identify variables associated with childhood stunting. For these multivariable analyses, we included all variables that were statistically significant at 10% in bivariate analysis into the model. We used the Hosmer-Lemeshow goodness-of-fit test to assess the performance of our logistic regression model. For all analyses, *p*-values less than 0.05 were considered to be statistically significant. We present the resulting odds ratios with their corresponding 95% confidence intervals. Statistical analyses were performed using Stata (version 12) [26] and R (version 3.3.2) [27].

## Results

### Trend in the prevalence of childhood stunting from 2000 to 2015

The prevalence of stunting among children under five in Rwanda was relatively stagnant between 2000 and 2010 but showed an overall decline nationally from 47.4% in 2000 to 38.3% in 2015 (*p*-value < 0.001) (Table 1).

**Table 1:** Prevalence of childhood stunting by selected variables, Rwanda DHS 2000–2015

	Year 2000				Year 2005				Year 2010				Year 2015			
	%	95%CI	N	P-value	%	95%CI	N	P-value	%	95%CI	N	P-value	%	95%CI	N	P-value
Overall	47.4	[45.7,49.1]	6287		51.9	[50.0,53.9]	3765		44.0	[42.1,45.9]	4076		38.3	[36.4,40.2]	3538	
Child age (in months)																
0–11	22.8	[20.5,25.4]	1592	0.000	26.2	[23.1,29.5]	847	0.000	20.5	[17.7,23.6]	777	0.000	18.2	[15.3,21.5]	760	0.000
5–23	51.1	[48.0,54.2]	1233		57.7	[54.0,61.3]	786		49.8	[46.0,53.6]	786		47.9	[44.3,51.5]	731	
24–35	58.9	[55.4,62.3]	1106		61.9	[58.1,65.5]	848		51.3	[47.8,54.9]	874		45.1	[41.5,48.7]	742	
36–47	56.7	[53.2,60.1]	1142		59.6	[55.6,63.4]	637		50.9	[47.5,54.3]	823		43.5	[39.7,47.4]	743	
48–59	56.7	[53.7,59.8]	1214		57.7	[53.4,61.8]	647		45.9	[42.3,49.6]	816		37.0	[33.0,41.3]	562	
Sex																
Girls	44.9	[42.9,47.0]	3140	0.000	50.4	[47.9,52.9]	1912	0.094	40.7	[38.2,43.2]	2023	0.000	33.9	[31.5,36.5]	1732	0.000
Boys	49.8	[47.6,52.0]	3147		53.5	[50.8,56.3]	1853		47.3	[44.9,49.6]	2053		42.5	[40.0,45.1]	1806	
Birth weight																
≥ 2.5 kg	37.2	[34.3,40.1]	1845	0.014	42.8	[39.3,46.2]	1146	0.007	39.8	[37.6,42.0]	2666	0.000	36.4	[34.5,38.3]	3119	0.000
< 2.5 kg (low)	50.0	[40.0,60.0]	136		63.3	[48.6,75.9]	52		59.3	[51.0,67.1]	149		55.7	[47.8,63.3]	172	
Maternal Age																
< 25	44.0	[40.7,47.2]	1248	0.006	48.4	[44.3,52.6]	674	0.201	39.7	[35.9,43.7]	729	0.002	34.9	[31.2,38.9]	666	0.338
25–34	46.6	[44.2,49.0]	3114		52.4	[49.8,55.1]	1950		43.0	[40.6,45.4]	2198		38.8	[36.4,41.3]	2000	
35–39	48.6	[44.9,52.4]	1069		54.6	[50.1,58.9]	597		47.6	[43.2,52.0]	653		39.7	[34.8,44.8]	549	
40–49	53.2	[49.4,57.0]	856		51.8	[47.5,56.0]	544		50.0	[45.2,54.9]	496		39.8	[34.2,45.6]	323	
Maternal education																
No education	52.5	[50.0,55.0]	1978	0.000	56.3	[52.7,59.8]	1008	0.000	50.7	[46.9,54.4]	772	0.000	47.4	[42.7,52.0]	496	0.000
Primary	47.5	[45.4,49.5]	3481		51.7	[49.4,54.0]	2400		44.7	[42.5,46.8]	2924		39.6	[37.5,41.8]	2546	
Secondary+	31.0	[27.3,35.1]	828		39.7	[33.4,46.5]	357		23.1	[18.6,28.4]	380		20.7	[16.9,25.2]	496	
Maternal smoking history																
No	46.9	[45.2,48.6]	5967	0.004	51.6	[49.6,53.6]	3647	0.036	43.7	[41.8,45.6]	4027	0.001	38.0	[36.2, 40.0]	3494	0.000
Yes	55.2	[50.0,60.3]	320		62.4	[52.5,71.4]	118		68.4	[53.3,80.5]	49		58.2	[42.4,72.5]	44	
Residence																
Urban	33.5	[29.5,37.9]	1403	0.000	38.3	[34.1,42.8]	744	0.000	27.1	[22.3,32.5]	546	0.000	25.9	[21.9,30.3]	768	0.000
Rural	49.8	[48.1,51.6]	4884		54.1	[52.0,56.2]	3021		46.2	[44.2,48.2]	3530		40.8	[38.7,42.9]	2770	
Birth order and preceding birth interval																
First	46.3	[43.0,49.5]	1221	0.036	50.2	[45.8,54.5]	630	0.007	36.4	[33.1,39.8]	1000	0.000	34.9	[31.8,38.1]	978	0.014
2–3 and < 24 months	48.7	[44.6,52.9]	706		54.7	[48.9,60.3]	374		42.8	[37.8,48.0]	415		40.0	[33.7,46.6]	262	
2–3 and > 24 months	43.7	[40.7,46.7]	1456		51.2	[47.5,54.8]	873		45.9	[42.8,49.1]	962		36.9	[33.9,40.0]	1174	
4+ and < 24 months	48.6	[44.4,52.9]	634		60.3	[54.9,65.4]	451		51.6	[45.8,57.4]	324		45.8	[36.8,55.0]	154	
4+ and > 24 months	49.4	[47.0,51.8]	2270		50.0	[47.2,52.7]	1437		46.7	[43.8,49.6]	1375		41.7	[38.4,45.0]	970	
Wealth index																
Highest	29.3	[26.2,32.5]	1252	0.000	36.6	[32.4,40.9]	719	0.000	25.5	[22.0,29.3]	708	0.000	21.6	[17.9,25.9]	672	0.000
Fourth	48.7	[45.3,52.0]	1218		51.4	[47.1,55.7]	791		39.5	[35.9,43.2]	776		29.9	[26.1,34.0]	597	
Middle	49.9	[46.6,53.1]	943		52.1	[48.3,55.8]	752		45.7	[41.6,49.9]	809		38.4	[34.4,42.5]	660	
Second	51.1	[47.7,54.4]	1093		55.3	[51.3,59.3]	765		50.6	[47.0,54.2]	890		46.0	[42.4,49.8]	744	
Lowest	51.4	[48.6,54.1]	1781		61.1	[57.4,64.8]	738		53.2	[49.4,57.0]	893		48.3	[45.0,51.8]	865	

### Bivariate analysis: prevalence of childhood stunting by household, maternal, and child characteristics

**Fig. 1:**
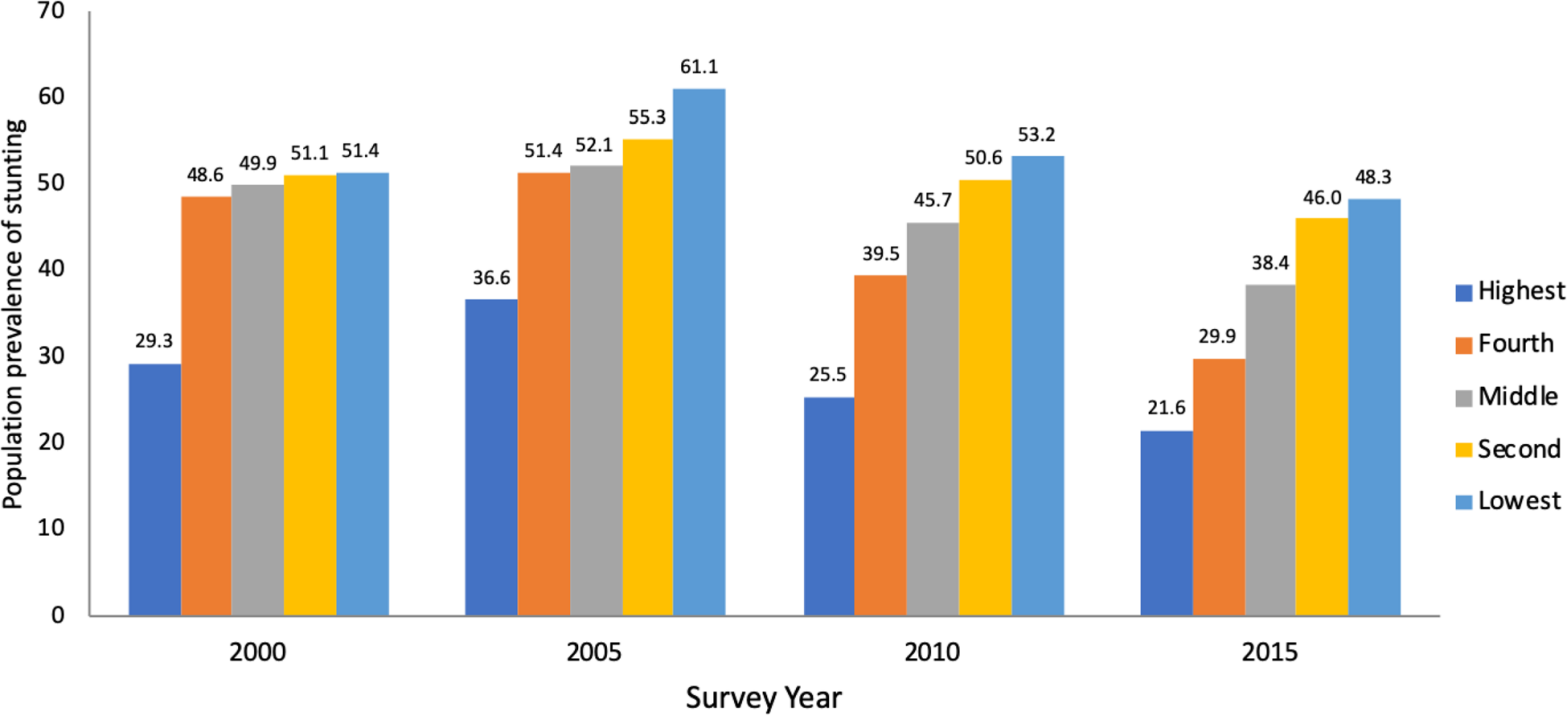
Trends in Prevalence of Childhood Stunting in Rwanda, by Wealth Quintile. Notes: Rwanda’s Demographic Health Survey (DHS) 2010 and DHS 2015 rely on the 2006 WHO standards whereas DHS 2000 & 2005 do not. For consistency, all of our calculations were conducted according to the 2006 standards. As such, our calculations use a different definition of malnutrition in 2000 and 2005 than the DHS publications do for those years.

The risk of stunting was associated with lower household economic status for all time periods. The prevalence of childhood stunting was persistently higher among families from the poorest wealth quintile, remaining relatively stable between 2000 and 2015, (51.4% vs. 48.3%, respectively); whereas, children in households in the highest wealth quintile demonstrated an overall decline during that same time period (29.3% vs. 21.6%). While stunting among children under five in the highest wealth index households increased from 29.3% in 2000 to 36.6% in 2005, it fell to 21.8% by 2015 (Fig. 1). Moreover, childhood stunting was more prevalent among those living in rural areas relative to urban areas (40.8% versus 25.9% in 2015, respectively).

Regarding maternal factors, the childhood stunting rate was significantly higher among families with poorly educated mothers across all years relative to households with a mother who had higher levels of education (*p*-value< 0.001) (Table 1). Childhood stunting was also significantly greater among households where the mother smokes relative to households with non-smoking mothers (p-value< 0.001), ranging from 55.2% in 2000 to 58.2% in 2015 for smoking households to 46.9% in 2000 to 39.6% in 2015 in non-smoking households.

With respect to individual characteristics, stunting was highest among children with a low birth weight (ranging from 50.0% in 2000 to 55.7% in 2015 and peaking at 63.3% in 2005) relative to those with normal-to-high birth weights (p-value< 0.01). For age, the prevalence of stunting was greatest among children aged 24–35 months (58.9% in 2000, 61.9% in 2005, 51.3% in 2010, and 45.1% in 2015) relative to younger children (p-value< 0.01). Further, stunting was greater among boys relative to girls (p-value<.0001 across all years) (Table 1).

### Multivariable analysis results: factors associated with childhood stunting

In Table 2, we present the results of our multivariable analysis to examine factors associated with childhood stunting. The models included the following variables: sex, child age, birth weight, maternal education, mother’s smoking history, place of residence, and wealth quintile.

**Table 2:** Odds ratios (ORs) and 95% confidence intervals (95%CIs) for factors associated with childhood stunting, Rwanda DHS 2000–2015

Parameters	Year 2000		Year 2005		Year 2010		Year 2015	
	OR	95%CI	OR	95%CI	OR	95%CI	OR	95%CI
Sex								
Girl	Ref		Ref		Ref		Ref	
Boy	1.35*	(1.01–1.86)	1.03	(0.77–1.38)	1.53***	(1.25–1.86)	1.51***	(1.30–1.76)
Child age								
6–11	Ref		Ref		Ref		Ref	
12–23	5.34***	(3.46–8.24)	4.16***	(2.60–6.66)	4.48***	(3.21–6.25)	3.48***	(2.56–4.71)
24–35	6.42***	(3.92–10.5)	3.65***	(2.29–5.82)	4.73***	(3.43–6.53)	3.53***	(2.63–4.76)
36–47	5.86***	(3.29–10.4)	3.84***	(2.33–6.33)	5.68***	(4.02–8.01)	3.15***	(2.27–4.37)
48–59	6.80***	(3.58–12.9)	2.75***	(1.65–4.61)	3.77***	(2.63–5.39)	2.35***	(1.71–3.23)
Birth weight								
≥2.5 kg	Ref		Ref		Ref		Ref	
< 2.5 kg (low)	2.57***	(1.31–5.05)	1.59	(0.74–3.40)	2.29***	(1.42–3.70)	1.77***	(1.23–2.56)
Maternal education								
No education	Ref		Ref		Ref		Ref	
Primary	0.61**	(0.40–0.94)	1.00	(0.68–1.47)	0.89	(0.68–1.14)	0.84	(0.68–1.05)
Secondary or Higher	0.35***	(0.20–0.60)	0.78	(0.47–1.31)	0.42**	(0.26–0.66)	0.48***	(0.34–0.69)
Maternal smoking history								
No	Ref		Ref		Ref		Ref	
Yes	0.93	(0.41–2.14)	4.93**	(1.33–18.3)	4.98**	(1.59–15.60)	1.67	(1.00–2.79)
Place of residence								
Urban	Ref		Ref		Ref		Ref	
Rural	1.53	(0.980–2.39)	1.44*	(1.01–2.06)	1.34	(0.94–1.91)	1.17	(0.86–1.60)
Wealth quintile								
Highest	Ref		Ref		Ref			
Fourth	0.86	(0.52–1.42)	1.93**	(1.25–2.99)	1.56**	(1.10–2.22)	1.23	(0.85–1.77)
Middle	1.27	(0.65–2.46)	1.74*	(1.07–2.82)	1.61**	(1.12–2.32)	1.74***	(1.22–2.49)
Second	1.38	(0.72–2.63)	2.07**	(1.26–3.40)	2.39***	(1.66–3.44)	2.35***	(1.64–3.35)
Lowest	1.13	(0.61–2.09)	1.73	(0.98–3.10)	2.68***	(1.86–3.87)	2.71***	(1.90–3.87)

*** *p* < 0.001, ** *p* < 0.01, * *p* < 0.05. Variables in models included sex, child age, birthweight, maternal education, mother’s smoking history, place of residence, and wealth quintile. Low birth weight for children was defined as less than 2.5 kg.

#### Household factors

Lower economic status was associated with childhood stunting, with children under 5 years of age in the poorest households (lowest wealth index) having significantly higher odds of stunting relative to those in the richest (highest wealth index) households (Table 2). Over time, this disparity between rich and poor appeared to increase; in 2000 and 2005 there was no significant difference in stunting rates among children below the age of 5 in the poorest households relative to the richest. However, children from the poorest households had 2.68 and 2.71 higher odds of stunting than children in the richest households in 2010 and 2015, respectively (*p*-value< 0.001).

#### Maternal factors

There is a consistent inverse relationship between maternal education level and childhood stunting: the more schooling a mother has, the less likely her child is to be stunted. Specifically, in 2000, children below the age of 5 whose mothers attended primary school had lower odds of stunting than children whose mother had no education (odds ratio (OR) = 0.61, *p*-value< 0.01); and children whose mothers had a secondary and higher level of education had lower odds of stunting than those with mothers who had no education (OR = 0.35, p-value< 0.001). While this disparity was not statistically significant in 2005, it was significant in 2010 and 2015.

#### Child factors

Children classified as having a low birth weight (less than 2.5 kg) had more than twice the odds of being stunted relative to those born with normal weight (OR = 2.57 in 2000 and OR = 1.77 in 2015). Similar to our bivariate results, the odds of stunting were greater after the first year of life: in 2015, children greater than 12 months of age tended to be an estimated 2.35 to 3.53 times more likely to experience stunting compared to children aged 12 months and below (*p*-values< 0.001). This represented a decrease from 2000 whereas older children were between 5.35 and 6.8 times as likely to experience stunting (*p*-values < 0.001). Additionally, the analysis showed that boys were generally more likely to be stunted than girls (Table 2).

## Discussion

This study identified a series of risk factors associated with childhood stunting in Rwanda. This analysis provides important evidence that malnutrition has persisted in spite of other improvements in the country’s economy and health indicators and provides policymakers with guidance regarding particular at-risk populations for this persistent scourge on childhood well-being. Specifically, we found that living in a rural area, lower maternal education level, being in the lowest household wealth quintile, maternal smoking, being male, having a low birth weight, and being older than 1 year of age are risk factors associated with childhood stunting in Rwanda.

Our findings build upon prior work examining stunting in Rwanda and provide a more comprehensive time trend analysis. One cross-sectional study leveraging the 2015 DHS to identify risk factors for stunting similarly found that male sex, increased child age, low household wealth index, low birth weight, and lower maternal education level were risk factors for stunting [28]. They also found that low maternal height and a history of not taking deworming medicine during pregnancy were risk factors for stunting, suggesting areas for further analysis of these trends over time [28]. A study focused on the northern province of Rwanda found that increased child age was a risk factor for stunting and that both exclusive breastfeeding as well as use of deworming tablets in the previous 6 months were protective factors [29]. Further, Kirk et al. (2017) found a striking 78.3% stunting prevalence rate among preterm/low birthweight children discharged from a hospital neonatal unit in rural Rwanda, which is nearly double the national stunting prevalence rate [30].

The associated risk factors for stunting among Rwandan children are comparable to those observed in other countries. In South Africa, one study found significantly higher levels of stunting among children under five from the poorest segment of society as compared to the richest; rural populations also demonstrated higher levels of stunting compared to their urban counterparts [31]. Additionally, a study on Indonesia found that severe stunting in children under the age of five was associated with low parental education, low household wealth index, child’s age, and male sex [32]. Furthermore, in Uganda, a study identified similar risk factors for stunting, including low family socioeconomic status and limited maternal education [33]. A study in Mexico corroborates this finding, demonstrating that a lower level of maternal education is associated with the risk of stunted children [34].

A 141-country review of population-based data from 1985 to 2011 showed that economic gains were systematically associated with lower stunting prevalence [35]. However, our study shows that a high stunting prevalence has persisted in Rwanda, despite a growing economy. One contributing factor might be that families from the lowest wealth quintile tend to have the highest proportion of families with more than 5 members (10.9% in 2000 to 18.7% in 2005) [16, 18]. This pattern, combined with the fact that the lowest quintile also experienced a stagnated prevalence in childhood stunting between 2000 and 2015, may help explain why a high prevalence of stunting is observed despite overall economic gains in the country, including a reduction in the proportion of the population living in poverty (from 58.9% in 2000 to 38.2% in 2015) [36]. In other words, as the poorest families – whose children are at the highest risk of stunting relative to others – grow in size, the rate of childhood stunting has remained stagnant. A growing disparity in stunting between the rich and poor demonstrates that the significant progress made within the highest wealth quintile has not reached the poorest members of society, which necessitates closer attention given Rwanda’s commitment to equity. Additionally, our findings suggest the importance of further broadening women’s access to higher education, increasing access to reproductive and postnatal care to reduce the burden of low birthweight, improving uptake of family planning services, ensuring access to clean drinking water and sanitation, and promoting the cessation/prevention of maternal smoking are necessary to adequately tackle stunting risk factors associated with maternal and antenatal health.

Policies to curb stunting in Rwanda are already underway to help children in the poorest sectors of the population, including the National Multisectoral Strategy to Eliminate Malnutrition (2010), the National Community Based Nutritional Protocol 2010, the District Action Plans to Eliminate Malnutrition (2011), and the Joint Action Plan to Fight Malnutrition (2013) [37]. Further, Rwanda’s free public education program for children younger than 18 years old and an extensive adult literacy program to address the limited education of current mothers, may also help to prevent the perpetuation of these disparities [38, 39]. Moreover, to increase food intake, there are school feeding programs, kitchen garden initiatives, the one-cow one-family program, and a distribution of livestock to the poorest families. It will be imperative to monitor the progress of these policies and initiatives to mitigate the burden of stunting among children in Rwanda. Further, there is evidence that enrollment in Rwanda’s health insurance scheme (*Mutuelles*)*,* which covers nutrition-related promotional and preventive services, could help combat stunting nationally [40].

In the long-term, a high level of stunting among children has implications for future economic security as they transition into adulthood, which can serve as a potential threat to Rwanda’s ability to achieve its Vision 2020 development goals, broadly defined as “macroeconomic stability and wealth creation to reduce aid dependency”, “structural economic transformation” and “creating a productive middle class and fostering entrepreneurship” [9]. Therefore, it is important to undertake additional research to help explain this discrepancy between national economic growth, health progress, and malnutrition. For example, although not directly examined in this paper, a study by Milman and colleagues suggested that countries devoting more resources to agriculture witness worse stunting levels than countries with a more diversified economy [41]. Since such a high proportion of Rwanda’s economy is generated from agricultural production, with agriculture, forestry, and fishing comprising 31% of the 2017 gross domestic product, this suggests the need for additional analyses to explore this potential association in the Rwanda context [42].

### Limitations

Though our study provides a comprehensive overview of the best population-based data available between 2000 and 2015, it has important limitations as a secondary analysis. The DHS data are cross-sectional; therefore, examining a temporal relationship between relevant risk factors and stunting is not possible. Further, since DHS data only includes families living in households, it omits potentially relevant, high risk groups such as homeless, refugee, hospitalized or otherwise institutionalized children, which may bias our results towards an underestimate of the burden of stunting. There are limitations in the wealth index as well; household income and wealth are not specifically measured by the DHS and the evaluation of wealth based on ownership of durable goods and household characteristics may not capture the complexity of resources available to individuals in a household. This metric serves purely as a proxy for socioeconomic status in our analysis. Additionally, adjusting scoring for households based on urban or rural residence was not introduced into the Rwanda DHS until 2015. Further, this analysis did not include all potentially relevant factors, such as psychosocial risk factors including maternal depression and intimate partner violence, as these were beyond the study’s scope.

## Conclusions

In spite of substantial country-wide economic gains over the past two decades, Rwanda has witnessed a relatively stagnant rate of childhood stunting since 2000, with only a marginal decrease in 2015. These findings can inform current programs underway and help to tailor future national strategies to make needed progress to reduce stunting among children in Rwanda. This will necessitate a multi-sectoral approach and one that reduces socioeconomic inequalities, dissolves rural and urban disparities, breaks barriers for women’s access to education, increases access to safe water and sanitation services, as well as improves uptake of reproductive, maternal, and postnatal care. Progressive policies and programs that support these changes can also advance the national equity agenda by improving the wealth distribution and averting unnecessary suffering among children and their families. These policies and strategies will support Rwanda in achieving its Vision 2020 goals and promoting economic advancement in the long-term.

## Data Availability

All data come from the Rwanda Demographic Health Surveys completed between 2000 and 2015 - https://dhsprogram.com/what-we-do/survey/survey-display-554.cfm. Additional information regarding the datasets used and analysed in this current study are available from the corresponding author on reasonable request.

## Abbreviations

DHS: Demographic and Health Survey
OR: Odds Ratio
SD: Standard Deviation
UNICEF: United Nations International Children’s Emergency Fund
WHO: World Health Organisation
